# Effect of Different Enzyme Treatments on Juice Yield, Physicochemical Properties, and Bioactive Compound of Several Hybrid Grape Varieties

**DOI:** 10.3390/molecules30030556

**Published:** 2025-01-26

**Authors:** Muhamad Alfiyan Zubaidi, Marta Czaplicka, Joanna Kolniak-Ostek, Agnieszka Nawirska-Olszańska

**Affiliations:** 1 Department of Fruit, Vegetable and Plant Nutraceutical Technology, Faculty of the Biotechnology and Food Science, Wrocław University of Environmental and Life Sciences, 51-630 Wrocław, Poland; muhamad.zubaidi@upwr.edu.pl (M.A.Z.); joanna.kolniak-ostek@upwr.edu.pl (J.K.-O.); 2 Department of Horticulture, Wroclaw University of Environmental and Life Sciences, 50-375 Wrocław, Poland

**Keywords:** enzyme treatment, grape juice, juice yield, polyphenols, antioxidant capacity

## Abstract

This study investigates the effects of four enzymatic treatments on the yield, physicochemical properties, and bioactive compounds of grape juices from two red (Golubok, Regent) and two white (Muscaris, Aurora) hybrid grape varieties. A total of 20 samples were prepared using four commercial enzyme formulations (Pectinex Ultra, Safizym Clear Plus, Safizym Press, and Rapidase color) applied at a concentration of 0.02% (*w/w*). The juices were evaluated for yield, total phenolic content, antioxidant capacities (ABTS, DPPH, FRAP), titratable acidity, turbidity, total soluble solids, and phenolic profile. The addition of enzymes significantly improved juice yield by 10% to 20%, with the effect varying depending on the type of enzyme and the variety of grapes. Pectinex Ultra was the most effective enzyme in reducing turbidity, while enzyme treatments had minimal impact on Brix levels and sugar concentration, which were primarily determined by the characteristics of each grape variety. The enzyme addition showed a minor influence on the titratable acidity of the juices, with slight increases observed in Muscaris, but the grape variety played a major role in determining the titratable acidity levels. Color parameters revealed that white grape juices (Muscaris and Aurora) were brighter than red varieties (Golubok and Regent). Additionally, enzyme treatments influenced the color, enhancing the red hues in red grape juices. Enzyme treatments also improved the antioxidant capacity of grape juices, especially in Aurora and Muscaris, although the effect on polyphenol content was more dependent on the variety of grapes, with red varieties showing higher levels of polyphenols than white varieties. These findings highlight the significant role of both enzyme treatments and grape variety in determining the quality and health-promoting properties of grape juice.

## 1. Introduction

Grape juice is widely recognized not only for its refreshing flavor but also for its rich content of bioactive compounds, particularly polyphenols, which have been associated with various health benefits, including antioxidant, anti-inflammatory, and cardioprotective effects [1]. These compounds, along with other phytochemicals present in grapes, make grape juice a potential functional beverage that can contribute to the prevention of chronic diseases [2]. However, the extraction of these valuable compounds and the optimization of the juice yield remain significant challenges in the production process, especially with hybrid grape varieties, which often have variable physicochemical properties [3].

Hybrid grape varieties are developed through interspecies crosses involving wild grape species and *Vitis vinifera*. These hybrids are typically more resistant to adverse climate conditions and fungal infections, making them suitable for cultivation in regions with challenging environments [4]. Additionally, hybrid varieties often exhibit high levels of bioactive compounds, such as polyphenols and flavonoids, which contribute to their antioxidant and health-promoting properties [5]. Although their sensory attributes may differ slightly from *Vitis vinifera*, their nutritional profile makes them an increasingly valuable resource for producing functional beverages and ensuring sustainable grape production.

To address these challenges, enzyme-assisted extraction has emerged as a promising technique to improve both the yield and the quality of grape juice [6]. Enzymes such as pectinases, cellulases, and hemicellulases are commonly used in fruit juice processing to break down cell walls, facilitate juice release, and improve phenolic compound extraction [7]. Enzyme-assisted extraction not only increases yield but also has the potential to increase the concentration of bioactive compounds, which is critical for producing high-quality functional beverages [8].

This study explores the effects of four different enzymatic treatments on grape juice processing from two white grape varieties (Muscaris and Aurora) and two red grape varieties (Golubok and Regent). Our objective is to assess the influence of these enzymes in enhancing the yield and improving the physicochemical properties of grape juice, including the total phenolic content, antioxidant capacities, titratable acidity, turbidity, total soluble solids, and the phenolic profile determined by means of UPLC. These parameters were selected for their significance in determining both the quality and the functional value of grape juice. By comparing the effects of enzymatic treatments against a control group (no enzyme treatment), we seek to determine the optimal enzymatic approach to maximize juice yield and bioactive compound content.

This research contributes to the development of functional food by providing information on the enzymatic processing of grape juice, focusing on improving its nutritional profile and health-promoting properties. Enzyme-assisted processing could play a role in the development of functional beverages that support the prevention of chronic diseases by improving the concentration of bioactive compounds in grape juice.

## 2. Results and Discussions

### 2.1. Yield

**Figure 1:**
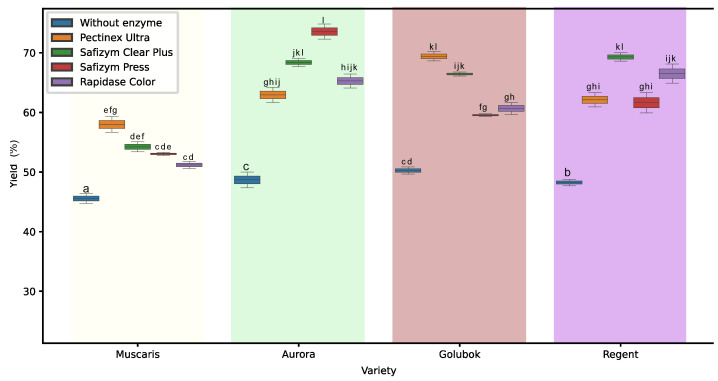
Box-and-whisker plot that illustrates the yield of grape juice yield in different grape varieties and enzyme treatments. The boxes followed by different letters are statistically different at *p* < 0.05, according to the Tukey post hoc test.

The data in Figure 1 represent the yield of grape juices obtained from four hybrid grape varieties treated with four enzymes. The results indicate that enzyme treatments significantly improved the yield in all grape varieties. An increase in yield is expected as the enzyme alters the walls of the grape cell [9]. On the other hand, without enzyme intervention, the juice yield was less than 50%, indicating the inefficiency of juice processing without any enzyme addition. A similar result was observed by Sharma et al. [10], stating that grape juice processing without enzyme addition results in a lower yield. Furthermore, Guo [11] observed a similar result, which was that enzyme treatments increased the yield of sea buckthorn juice.

Among the grape varieties, Aurora demonstrated the highest juice yield, ranging from 49.92% to 73.57%, while Muscaris exhibited the lowest juice yield, ranging from 45.42% to 57.98%. The difference in juice yield can be attributed to the Brix levels of the grape varieties. Muscaris, with the highest Brix level of 21.50, had the lowest yield due to its higher sugar content, which is correlated with a lower moisture content [12]. On the contrary, Aurora had the lowest Brix level, indicating a higher moisture content, leading to a higher yield. The correlation between Brix levels, moisture content, and juice yield is consistent with the findings of Zubaidi et al. [13], who observed that grapes with higher sugar content had a lower yield.

Furthermore, enzyme selection played an important role in determining the juice yield of each variety. For example, Pectinex Ultra produced the highest juice yields for the Muscaris and Golubok varieties, achieving yields of 57.98% and 69.41%, respectively. On the contrary, Safizym Press was most effective when added to the Aurora variety, resulting in a yield of 73.57%, while Safizym Clear Plus produced the highest juice yield for the Regent variety, reaching 69.30%. Therefore, selecting the appropriate enzyme based on the specific characteristics of the grape variety can significantly improve juice yield.

### 2.2. Total Soluble Solid and Turbidity

**Figure 2:**
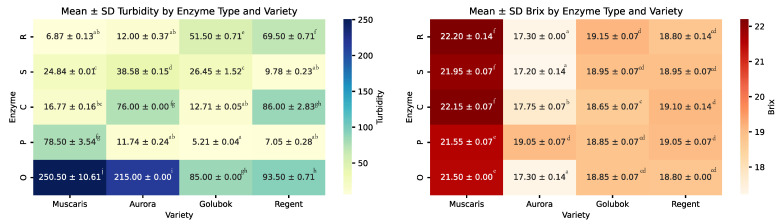
Heatmaps showing the mean ± standard deviation (SD) for turbidity (**left**) and Brix (**right**) across different enzyme treatments (O: without enzyme, P: Pectinex Ultra, C: Safizym Clear Plus, S: Safizym Press, R: Rapidase color) and grape varieties. Values followed by different letters are statistically different at *p* < 0.05, according to the Tukey post hoc test.

The turbidity and total soluble solids of the grape juice samples are represented in Figure 2. Turbidity is a measure of the cloudiness of juices caused by the presence of suspended particles. The higher the turbidity, the more abundant the particle [14]. The turbidity in grape juice plays an important role because it gives insight into the quality of the juice and consumer acceptance.

In general, the addition of enzymes effectively reduced turbidity in all grape varieties. However, the suitability of each enzyme varies depending on the specific grape variety, as not all enzymes are equally effective in reducing the turbidity for each grape variety. For example, the addition of the Safizym Clear Plus enzyme significantly reduced Muscaris turbidity, reducing it from 250.50 NTU to 16.77 NTU. However, the same enzyme had minimal impact on Regent, where the turbidity decreased slightly from 93.50 NTU to 85.00 NTU.

Pectinex Ultra was the most effective enzyme, reducing the turbidity to less than 15.00 NTU for all grape varieties except Muscaris. On the other hand, while Safizym Press was not as powerful as Pectinex Ultra in reducing turbidity, it reduced to below 30.00 NTU regardless of grape variety.

Furthermore, Safizym Clear Plus significantly reduced turbidity to below 17.00 NTU for Muscaris and Golubok varieties but was less effective for Aurora and Regent. Similarly, Rapidase color reduced turbidity to less than 15.00 NTU for Muscaris and Aurora but did not achieve the same level for Golubok and Regent. The results of these measurements indicate that the effectiveness of enzymes in decreasing turbidity is significantly dependent on the characteristics of the grape variety.

The Brix level indicates the sugar content of the grape juices; the higher the Brix level, the higher the sugar content. In general, enzymes did not significantly affect the Brix value of the juice samples. This indicates that the sugar in the grape was perfectly released during pressing, even without any enzyme addition. Therefore, enzymatic breakdown did not help to release additional sugar since all of the sugar had already been extracted.

Across all grape varieties, the Brix level remained constant even when enzymes were added during the maceration process. Muscaris juices had the highest Brix level, ranging from 21.50 to 22.20. On the contrary, Aurora exhibited the lowest Brix level, ranging from 17.20 to 19.05. The results highlight the importance of selecting the desired Brix level of the raw material before processing, as processing itself cannot significantly modify the Brix level of the juices.

### 2.3. Titratable Acidity

**Figure 3:**
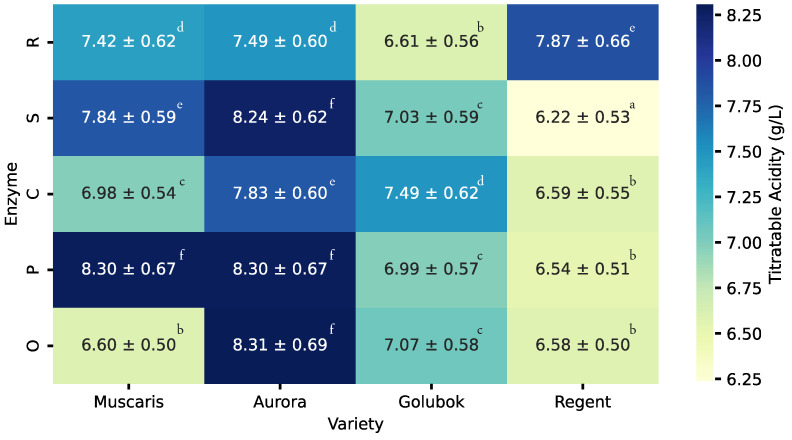
Heatmaps showing the mean ± standard deviation (SD) of titratable acidity (g/L) of different enzyme treatments (O: without enzyme, P: Pectinex Ultra, C: Safizym Clear Plus, S: Safizym Press, R: Rapidase color) and grape varieties. Values followed by different letters are statistically different at *p* < 0.05, according to the Tukey post hoc test.

The heatmap in Figure 3 illustrates the titratable acidity across different grape varieties and enzyme treatments. All juices obtained from hybrid grape varieties generally exhibited titratable acidity levels ranging from 6.22 g/L to 8.31 g/L. This range indicates that all juice samples possessed a balanced acidity, which is ideal for grape juices, as it provides a harmonious taste with moderate sourness and sweetness [15].

Among the varieties, Aurora grape juices consistently demonstrated the highest titratable acidity, ranging from 7.49 to 8.31 g/L. On the contrary, Regent variety juices showed the lowest acidity, consistently below 6.59 g/L, except when treated with the Rapidase color enzyme. Meanwhile, Golubok and Muscaris exhibited moderate acidity ranging from 6.60 g/L to 8.30 g/L and 6.61 g/L to 7.49 g/L, respectively.

The addition of enzymes did not consistently increase or decrease the titratable acidity of the resulting juices. For example, the acidity of the juice obtained from the Aurora variety was significantly reduced by the addition of Safizym Clear and Rapidase color enzymes, while it remained relatively stable with the addition of Pectinex Ultra and Safizym Press enzymes. Similarly, the Golubok variety showed minimal changes in acidity with enzyme addition, except when Safizym Clear was applied. For the Regent variety, the addition of enzymes did not generally have a significant effect, although Rapidase color increased the acidity to 7.87 g/L. In contrast, the Muscaris variety exhibited significant increases in titratable acidity regardless of the enzyme types. These findings suggest that targeted enzyme applications can effectively modulate titratable acidity levels based on the grape variety and desired product characteristics.

### 2.4. Color Parameters

**Figure 4:**
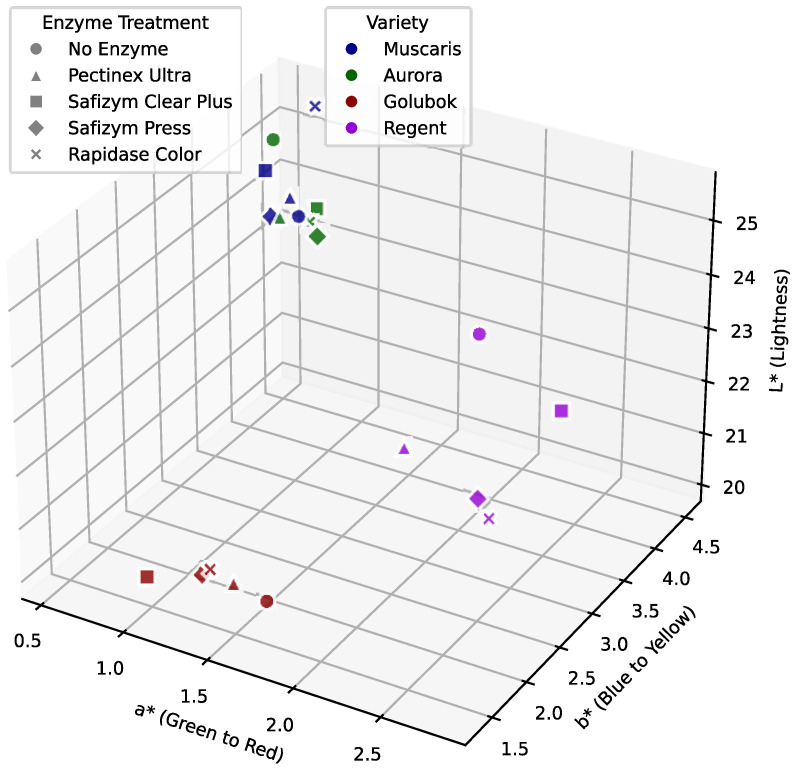
CIELab 3D color plot of grape juice samples.

Figure 4 shows the 3D pot of the CIE Lab color measurements. The juices obtained from the white grape varieties (Muscaris and Aurora) generally had higher L* values ranging from 23.77 to 25.48, indicating that the juices were brighter. For example, the sample MR and AO had the highest L* value, 25.48 and 25.27, respectively. In contrast, the red grape varieties (Golubok and Regent) had a lower L* value ranging from 20.07 to 23.43. Juice from the Golubok variety juice, in particular, had the lowest L * value, which is typical for darker red juices.

The red grape varieties (Golubok and Regent) exhibited higher *a values, indicating a more pronounced red hue in the juice. Regent (R) *a values range from 2.03 to 2.81, and Golubok (G), from 1.27 to 2.81. However, the white grape varieties (Muscaris and Aurora) had lower *a values, indicating less red. The *a values for Muscaris (M) range from 0.55 to 0.88, and for Aurora (A), from 0.64 to 0.93.

Furthermore, juices from red grape varieties tended to have lower *b values, suggesting a blueish color ranging from 1.35 to 1.96. In contrast, the juices from the white grape varieties had higher *b values, ranging from 3.86 to 4.53, indicating a yellowish color.

Furthermore, the enzyme treatment also influenced the color change in the grape juices, particularly in terms of the *a and *b values. For instance, Pectinex Ultra (P) tended to push the *a values higher, particularly in Regent (RP, RC) and Golubok (GP), indicating a shift toward more red hues. Furthermore, Safizym Clear Plus (C) appeared to result in slightly lower *a values for Muscaris (MC) and Aurora (AC), possibly resulting in a more neutral or less intense color. Rapidase color (R) appears to enhance the *a and *b values, resulting in deeper color shades in red grapes such as Regent (RR) and Golubok (GR).

### 2.5. Sugars

**Figure 5:**
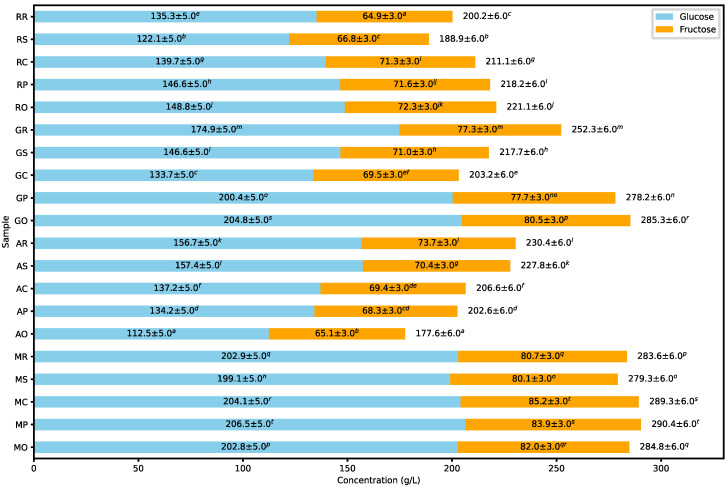
Bar chart showing the mean ± standard deviation (SD) of sugar concentration (g/L) of different enzyme treatments (O: without enzyme, P: Pectinex Ultra, C: Safizym Clear Plus, S: Safizym Press, R: Rapidase color) and grape varieties (M: Muscaris, A: Aurora, G: Golubok, R: Regent). Values followed by different letters are statistically different at *p* < 0.05, according to the Tukey post hoc test.

The sugar content of the grape juices was analyzed using HPLC to identify and quantify the sugar in the juices. Figure 5 shows that grape juices contain only glucose and fructose and no other types of sugar. The finding was similar to what Dutra et al. [16] observed in grape juices, which was that grape juices contain only glucose and fructose. Glucose concentrations are higher than fructose, ranging from 112.5 g/L to 204.8 g/L and 64.9 g/L to 85.2 g/L, respectively.

Among the grape varieties, the juice obtained from Muscaris varieties generally had a higher sugar content, ranging from 279.3 g/L to 290.4 g/L. On the contrary, the juice obtained from the Aurora variety had the lowest sugar content, ranging from 177.6 g/L to 230.4 g/L. The finding aligns with the Brix level, which shows Muscaris had the highest Brix level, indicating the highest sugar content, and reversely, Aurora, which had the lowest Brix value, exhibited the lowest sugar content.

In particular, enzyme addition does not significantly increase the sugar concentration of juices. For instance, in the Golubok and Regent varieties, enzyme treatments did not significantly increase the total sugar content; in fact, the values were lower than samples without enzyme treatments, 285.3 g/L and 221.1 g/L, respectively. Furthermore, the linear correlation analysis of grape varieties with sugar content exhibited a lower value of 0.15, while the correlation between enzyme addition and sugar content was lower than 0.02. This indicates that the grape variety had a minor influence on sugar concentrations, while enzyme treatments had virtually no effect on the sugar concentration of grape juices. This finding aligns with the observation by Puzovic et al. [17], who noted that the sugar content is primarily determined by the initial properties of grape juice.

### 2.6. Total Polyphenol Content and Antioxidant Capacity

**Figure 6:**
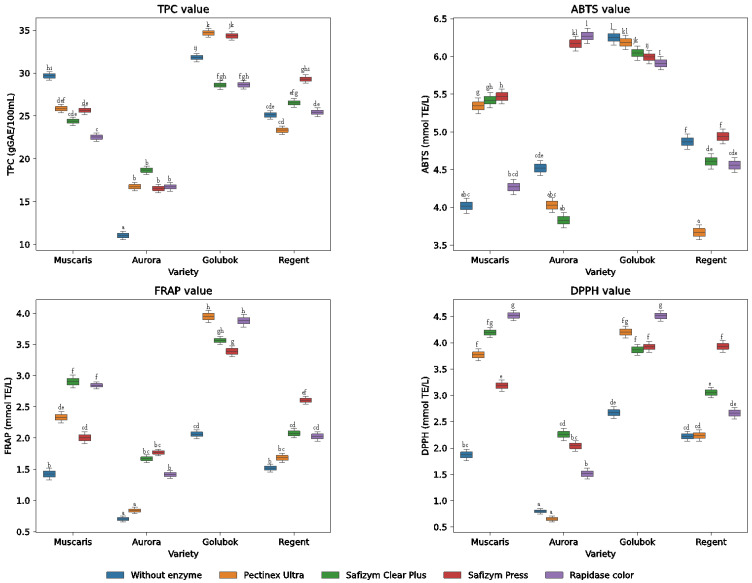
Box-and-whisker plot representing the total polyphenolic compounds (TPC) and antioxidant capacity measured by ABTS, FRAP, and DPPH methods in grape juices across different grape varieties and enzyme treatments. The boxes followed by different letters are statistically different at *p* < 0.05, according to the Tukey post hoc test.

The data presented in Figure 6 exhibited total polyphenol content (TPC) and antioxidant capacity (AC), measured by ABTS, FRAP, and DPPH assays. The juices from red grape varieties generally exhibited higher TPC values than those from white grape varieties. For example, the Golubok variety consistently demonstrated TPC values greater than 29 gGAE/100 mL in all treatments, while the Aurora variety consistently had TPC values lower than 20 gGAE/100 mL.

A study by Paixão [18] found a positive correlation between antioxidant capacity and total phenolic content, suggesting that samples with higher TPC values tend to exhibit greater antioxidant capacity. For example, the Golubok variety, with the highest TPC, also showed the highest antioxidant capacity in all measurement methods. On the contrary, the Aurora variety, with the lowest TPC, also exhibited the lowest antioxidant capacity in all methods.

Regarding the antioxidant capacity measured by ABTS, the Golubok variety juices exhibited the highest values, ranging from 6.0 to 6.35 mmol TE/L. On the contrary, without enzyme addition, the Aurora variety had the lowest ABTS value at 4.62 mmol TE/L. Interestingly, when enzyme treatments such as Safizym Press or Rapidase color were added, the Aurora ABTS value increased significantly to 6.27 mmol TE/L and 6.37 mmol TE/L, respectively. A similar increase was observed in Muscaris and Regent varieties juices, but enzyme treatment did not significantly affect the ABTS value in Golubok juices.

Golubok juices showed the highest antioxidant capacity for FRAP values, ranging from 3.48 to 4.05 mmol TE/L, while Aurora juices had the lowest FRAP values, ranging from 0.75 to 1.77 mmol TE/L. A similar trend was observed for the DPPH assay, with Golubok juices showing the highest values and Aurora the lowest. Interestingly, when the Rapidase color enzyme was added, Muscaris juices showed an antioxidant capacity greater than Golubok, reaching 4.62 mmol TE/L.

These results highlight the significant impact of enzyme addition in improving the antioxidant capacity of grape juices. Similar findings were reported by Guler [19] and Rinaldi [20] in studies on grape and pomegranate juices, underscoring the potential of enzyme treatments to improve antioxidant capacity.

**Figure 7:**
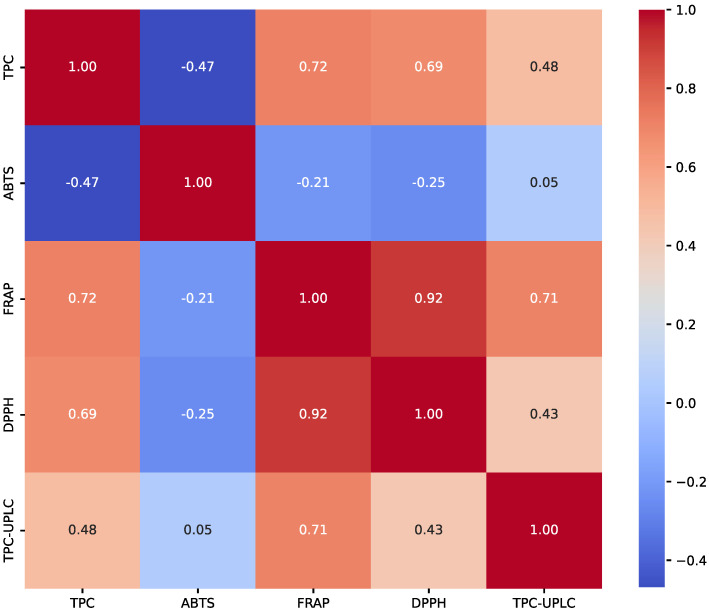
The correlation matrix of the five assays (TPC, ABTS, FRAP, DPPH, and TPC-UPLC).

Additionally, Figure 7 presents a correlation matrix illustrating the relationships between total polyphenol content, determined by the Folin–Ciocalteu method and UPLC, and antioxidant activities measured using the ABTS, FRAP, and DPPH assays. The data show that most parameters exhibited positive correlations. The strongest correlation was observed between FRAP and DPPH (0.92). Similarly, TPC and FRAP show a high positive correlation (0.72). However, ABTS displayed negative correlations with most assays, which may reflect differences in how this assay evaluates antioxidant capacity. Zubaidi et al. [13] reported a similar observation that the ABTS assay had the lowest correlation coefficients among antioxidant evaluation methods.

### 2.7. Polyphenolic Compound

Table 1 presents the polyphenolic content in grape juice samples from various red and white grape varieties. The data show that red grape varieties, Golubok and Regent, consistently exhibit higher polyphenol content than white grape varieties such as Muscaris and Aurora. Specifically, Golubok showed the highest total polyphenol content, ranging from 2440.76 g/L to 3941.02 g/L, while the white grape Aurora had the lowest, ranging from 386.46 g/L to 588.06 g/L. Furthermore, anthocyanins, a subclass of polyphenols, were only detected in red grape varieties, underscoring the contribution of these compounds to the total polyphenol content.

Among the samples, GR (Golubok with Rapidase color enzyme) had the highest polyphenol content in terms of all determined groups of polyphenol compounds: phenolic acids (722.92 g/L), flavonols (714.83 g/L), flavan-3-ols (2087.92 g/L), and anthocyanins (415.35 g/L). On the contrary, AO (Aurora without enzyme) recorded the lowest levels in all polyphenolic compounds, with phenolic acids (117.88 g/L), flavonols (16.55 g/L), and flavan-3-ols (252.03 g/L).

A closer examination of the trends reveals that the addition of enzymes increased the polyphenolic content in all grape varieties. For example, sample RO (Aurora without enzyme) had a total polyphenol content of 700.59 g/L, which increased to a range of 855.90 g/L to 1168.66 g/L with enzyme treatment. Similarly, in the Muscaris variety, the flavonol content rose from 59.02 g/L (without enzyme) to 196.14 g/L (with enzyme). Enzymes have the ability to break down cell wall structures, facilitating the release of more polyphenolic compounds from grapes [21].

However, while enzyme treatment positively affects polyphenol levels, grape variety plays a more significant role in determining the total polyphenol content. This is evident from the correlation analysis, which found a linear correlation coefficient of 0.13 between enzyme addition and total polyphenol content, compared to a much higher 0.56 between grape variety and total polyphenol content. This suggests that the intrinsic properties of the grape variety are the dominant factor in determining polyphenol levels rather than the processing conditions.

These findings are consistent with the conclusions of Dal Magro et al. [22], who observed that raw material (grape variety) has a greater impact on polyphenol content than the processing method. Although the addition of enzymes can enhance polyphenol extraction, the natural composition of the grape variety is the key determinant of the polyphenolic content in the juice.

## 3. Materials and Methods

### 3.1. Grapes

Two white grape varieties, Muscaris (M) and Aurora (A), and two red varieties, Golubok (G) and Regent (R), were harvested from the Research and Teaching Station of the Department of Horticulture at the University of Environmental and Life Sciences, located in the village of Samotwór, near Wroclaw in Lower Silesia Province, Poland. Table 2 provides details on the characteristics of each cultivar.

### 3.2. Reagents, Enzyme, and Standards

The standards were acquired from Extrasynthese (Lyon Nord, France) and include various quercetin compounds (quercetin-3-rutinoside, quercetin-3-glucuronide, quercetin-3-galactoside, quercetin-3-glucoside, quercetin-3-rhamnoside), kaempferol derivatives (kaempferol-3-*O*-rutinoside, kaempferol-3-*O*-galactoside, kaempferol-3-*O*-glucoside), caffeic acid, caftaric acid, coutaric acid, fertaric acid, isorhamnetin 3-glucoside, dihydroquercetin-3,5-rhamnoside, dihydrokaempferol-3-glucoside, procyanidins (trimer, B2, tetramer), (-)-epicatechin, (-)-epicatechin 3-gallate, petunidin-3-*O*-glucoside, malvidin-3-*O*-glucoside, and several anthocyanin derivatives in acetylated and coumaroylated forms. ABTS, gallic acid, FeCl_3_, Trolox, and the solvents were obtained from Sigma-Aldrich (Steinheim, Germany). The pectinase enzyme, Pectinex Ultra SP-L, was obtained from Novozymes in Copenhagen, Denmark, while Safizym Clear Plus, Safizym Press, and Rapidase color were provided by DSM in Delft, The Netherlands.

### 3.3. Juice Processing

The grapes were processed immediately on the day of harvest. Grapes were destemmed to remove stems and manually crushed. Enzymes were added at a concentration of 0.02% (*w/w*), using four types: Pectinex Ultra SP-L (P), Safizym Clear Plus (C), Safizym Press (S), and Rapidase color (R). For control, a sample of each variety was processed without the addition of enzymes (O). The grape mash was macerated at 40 °C for 90 min. After maceration, the mixture was pressed using a hydraulic press and filtered to obtain clear juice. The juices were frozen at −20 °C until further analysis. The varieties and treatments applied to the juices are detailed in Table 3.

### 3.4. Total Soluble Solids, Titratable Acidity, and Turbidity

Total soluble solids were analyzed using a PAL-1 refractometer (Atago, Bellevue, WA, USA). Measurements were made in duplicate and reported in °Brix.

Titratable acidity (TA) was assessed through titration using an Orion Star T910 titrator (Thermo Fisher Scientific, Beijing, China), following the Polish standard PN-90/A-75101/04 (1999) [23]. A 0.1 N NaOH solution was gradually added to the juice samples until a pH of 8.1 was reached, the process being monitored by an automated pH titration system. The analysis was duplicated, with results expressed as grams of tartaric acid per 100 mL.

The turbidity of the juice was determined using a Turbiquant 3000 T turbidimeter (Merck, Darmstadt, Germany). This measurement was conducted in duplicate and recorded in NTU units.

### 3.5. Color (L *a *b)

The color parameters of the grape juices were evaluated using a Color Quest XE Hunter Lab colorimeter (Reston, VA, USA). The device utilized the CIE standard Illuminant D65 with a 10° observer angle. Measurements were performed in triplicate, and results were expressed in the L *a *b color space, representing lightness (L), red–green axis (*a), and yellow–blue axis (*b).

### 3.6. Sugar Analysis

The sugar extraction for the analysis was performed following the method outlined by Oszmiański et al. [24]. The chromatographic analysis utilized a Merck-Hitachi L-7455 liquid chromatograph equipped with an evaporative light scattering detector (ELSD, Polymer Laboratories PL-ELS 1000) and an L-7100 quaternary pump, integrated with the D-7000 HSM Multisolvent Delivery System (Merck-Hitachi, Tokyo, Japan). The mobile phase consisted of acetonitrile and water in a ratio of 75:25 (*v/v*). The system also included an L-7200 autosampler and a Prevail Carbohydrate ES HPLC Column-W (250 mm × 4.6 mm, 5 µm) (Alltech, Nicholasville, KY, USA).

Calibration curves with a coefficient of determination (R^2^ = 0.9999) were prepared for glucose, fructose, sorbitol, and sucrose. All measurements were performed in duplicate, and the results were reported as grams per liter of grape juice.

### 3.7. Total Phenolic Content

The total polyphenolic content was assessed in 70% methanol extracts (*v/v*) using the Folin–Ciocalteu method, measurements were taken on a Synergy H1 spectrophotometer (BioTek Instruments Inc., Winooski, VT, USA). Each measurement was carried out in quadruplicate, and the results were expressed as grams of gallic acid equivalent (GAE) per 100 mL of juice.

### 3.8. Antioxidant Capacity

The extraction of samples to assess antioxidant capacity and polyphenol content followed the method outlined by Kolniak-Ostek [25]. Antioxidant capacity in the grape juice was measured using ABTS, FRAP, and DPPH assays on a Synergy H1 spectrophotometer (BioTek Instruments Inc., Winooski, VT, USA), based on protocols by Re et al. [26] for ABTS, Benzie and Strain [27] for FRAP, and Yen and Chen [28] for DPPH. Measurements were carried out in quadruplicate, and results were reported as mmol Trolox equivalents (TE) per liter of juice.

### 3.9. Identification and Quantification of Polyphenols

The polyphenols in the samples were identified and quantified using UPLC-PDA-FL (Waters Corp., Milford, MA, USA and Dublin, Ireland). Sample preparation and analysis followed the method by Nawirska-Olszańska et al. [29]. A 5 μL sample was injected via an autosampler (BEH C18 column, 2.1 × 100 mm, 1.7 µm; Waters Corp., Dublin, Ireland) at 30 °C with gradient elution (0–12 min, 98–65% A; 12.1–13.5 min, 0% A; 13.6–15 min, 98%) using solvent A (0.1% formic acid in water) and solvent B (0.1% formic acid in acetonitrile). Separation parameters for the mass spectrometer were based on the method by Kolniak-Ostek and Oszmiański [30]. Polyphenol identification was determined by comparing retention times (Rt) with those of standards, and quantification was performed using calibration curves for selected pure compounds.

### 3.10. Statistical Analysis

Statistical analyses were performed with Statistica 14 (Statsoft, Tulsa, OK, USA). Data visualizations were performed using Python in Jupyter Notebook with Pandas 1.5.3 (by Anaconda, Inc., Austin, TX, USA) within the Anaconda environment. Factorial analysis of variance (ANOVA) with Tukey’s HSD post hoc test (*p* < 0.05) was performed to compare the results.

## 4. Conclusions

The results highlight the influence of enzyme treatment on the yield, physicochemical properties, and bioactive compounds of grape juices from different grape varieties. Enzyme treatments significantly increase juice yield by 10% to 20%, depending on the type of enzyme and grape variety. Among the enzymes tested, Pectinex Ultra is the most effective in reducing turbidity. However, enzyme treatments have minimal effect on total soluble solid and sugar content, which are determined primarily by the inherent characteristics of grape varieties.

Titratable acidity showed only a slight change with enzyme treatments, with a minor increase observed in Muscaris juice. In contrast, grape variety played a more significant role in determining acidity levels, with varieties such as Aurora exhibiting higher acidity and Regent showing lower acidity. Color measurements show that white grape juices (Muscaris, Aurora) are brighter (higher L* values) than red grape juices (Golubok, Regent). Enzyme treatments influenced color, particularly enhancing red hues in red grape varieties. Additionally, enzyme treatments improved the antioxidant capacity of grape juices, especially in Aurora and Muscaris. However, the effect on the polyphenolic content depended more on the grape variety than on enzyme treatment alone.

The results demonstrate the potential of enzyme-assisted processing to enhance the quality, nutritional value, and marketability of grape juices, offering valuable insights for the food industry and supporting the development of functional beverages with health-promoting properties.

## Figures and Tables

**Table 1:** The polyphenol content of the grape juice samples (mg/L of juice) ^‡^.

Samples	Phenolic Acids	Flavonols	Flavan-3-ols	Anthocyanins	Total
MO	145.03 ± 7.82 ^ab^	59.02 ± 9.21 ^ab^	387.11 ± 0.36 ^de^	nd	591.17 ± 1.74 ^abc^
MP	140.37 ± 5.84 ^ab^	146.52 ± 15.38 ^abc^	391.63 ± 0.45 ^ded^	nd	678.52 ± 9.98 ^bcd^
MC	139.72 ± 5.54 ^ab^	196.14 ± 16.50 ^abc^	397.14 ± 0.60 ^e^	nd	733.01 ± 11.55 ^cde^
MS	122.38 ± 5.05 ^a^	118.80 ± 13.49 ^ab^	338.42 ± 0.56 ^bcde^	nd	579.60 ± 9.00 ^abc^
MR	118.68 ± 7.10 ^a^	153.54 ± 14.74 ^abc^	326.32 ± 1.00 ^bcd^	nd	598.54 ± 8.64 ^abc^
AO	117.88 ± 10.19 ^a^	16.55 ± 5.52 ^a^	252.03 ± 0.22 ^a^	nd	386.46 ± 4.45 ^a^
AP	117.30 ± 10.70 ^a^	53.77 ± 6.11 ^ab^	298.92 ± 0.52 ^abc^	nd	469.99 ± 4.07 ^ab^
AC	167.87 ± 10.48 ^ab^	58.57 ± 7.62 ^ab^	349.95 ± 0.34 ^bcde^	nd	576.39 ± 3.19 ^abc^
AS	165.20 ± 9.27 ^ab^	55.73 ± 7.94 ^ab^	367.13 ± 0.35 ^cde^	nd	588.06 ± 1.68 ^abc^
AR	119.56 ± 7.37 ^a^	42.00 ± 7.04 ^ab^	286.70 ± 0.27 ^ab^	nd	448.26 ± 0.60 ^a^
GO	537.50 ± 21.92 ^d^	274.34 ± 88.69 ^bcd^	1406.52 ± 1.39 ^i^	222.39 ± 0.54 ^d^	2440.76 ± 64.84 ^g^
GP	623.70 ± 23.14 ^e^	369.38 ± 30.52 ^cd^	1629.59 ± 77.57 ^j^	298.20 ± 63.96 ^e^	2920.87 ± 134.15 ^h^
GC	716.99 ± 28.21 ^f^	666.49 ± 143.38 ^ef^	2002.08 ± 0.28 ^k^	355.41 ± 2.10 ^e^	3740.96 ± 112.78 ^i^
GS	597.35 ± 23.03 ^de^	479.35 ± 106.42 ^de^	1673.32 ± 0.43 ^j^	311.35 ± 3.27 ^e^	3061.36 ± 79.68 ^h^
GR	722.92 ± 27.53 ^f^	714.83 ± 140.50 ^f^	2087.92 ± 0.07 ^l^	415.35 ± 3.52 ^f^	3941.02 ± 109.52 ^i^
RO	137.18 ± 7.05 ^a^	36.52 ± 13.12 ^a^	509.54 ± 2.12 ^f^	17.34 ± 0.01 ^a^	700.59 ± 8.20 ^cd^
RP	172.64 ± 9.54 ^ab^	75.70 ± 27.40 ^ab^	580.71 ± 2.05 ^g^	26.85 ± 0.31 ^ab^	855.90 ± 19.59 ^de^
RC	173.90 ± 10.08 ^ab^	86.63 ± 25.60 ^ab^	598.45 ± 1.78 ^g^	82.49 ± 1.06 ^b^	941.47 ± 16.25 ^e^
RS	201.07 ± 11.33 ^b^	126.28 ± 33.00 ^ab^	739.80 ± 1.97 ^h^	148.60 ± 0.90 ^c^	1215.75 ± 22.74 ^f^
RR	363.93 ± 27.53 ^c^	87.20 ± 59.58 ^ab^	670.14 ± 0.06 ^h^	47.39 ± 1.05 ^ab^	1168.66 ± 33.04 ^f^

^‡^ value ± SD values are means of two repetitions; mean values followed by different letters are statistically different at *p* < 0.05 within the same column according to the Tukey post hoc test. O: without enzyme, P: Pectinex Ultra, C: Safizym Clear Plus, S: Safizym Press, R: Rapidase color, M: Muscaris, A: Aurora, G: Golubok, and R: Regent.

**Table 2:** Characteristics of the hybrid cultivars used in the study.

Cultivars	Parents	Skin Color	Flesh Color
Muscaris	Muskateller × Solaris	Green	White
Aurora	Seibel 788 × Seibel 29	Green	White
Golubok	Severnyy X pollen from different varieties: 40 Let Okyabrya, Odesskiy Ranniy and No 1-17-54 (Alicante Bouschet and Cabernet Sauvignon)	Dark	Dark
Regent	Diana × Chambourcin	Dark	White

**Table 3:** The grape juice samples.

Sample	MO	MP	MC	MS	MR	AO	AP	AC	AS	AR	GO	GP	GC	GS	GR	RO	RP	RC	RS	RR
**Variety**	Muscaris					Aurora					Golubok					Regent				
**Grape-type**	White					White					Red					Red				
**Enzyme**	O	P	C	S	R	O	P	C	S	R	O	P	C	S	R	O	P	C	S	R

## Data Availability

The data supporting the reported results were generated from laboratory measurements conducted during the study. Due to their specific nature and context of generation, these data are not publicly available.

## References

[1] Folts J.D., Buslig B.S., Manthey J.A. (2002). Potential Health Benefits from the Flavonoids in Grape Products on Vascular Disease. Flavonoids in Cell Function.

[2] Blumberg J.B., Vita J.A., Chen C.-Y.O. (2015). Concord Grape Juice Polyphenols and Cardiovascular Risk Factors: Dose-Response Relationships. Nutrients.

[3] Czaplicka M., Parypa K., Szewczuk A., Gudarowska E., Rowinśka M., Zubaidi M.A., Nawirska-Olszańska A. (2022). Assessment of Selected Parameters for Determining the Internal Quality of White Grape Cultivars Grown in Cold Climates. Appl. Sci..

[4] Kapusta I., Cebulak T., Oszmiański J. (2018). Characterization of Polish Wines Produced from the Interspecific Hybrid Grapes Grown in South-East Poland. Eur. Food Res. Technol..

[5] Kowalczyk B., Bieniasz M., Kostecka-Gugała A. (2022). The Content of Selected Bioactive Compounds in Wines Produced from Dehydrated Grapes of the Hybrid Variety ‘Hibernal’as a Factor Determining the Method of Producing Straw Wines. Foods.

[6] Jiao S., Li Y., Wang Z., Sun-Waterhouse D., Waterhouse G.I.N., Liu C., Wang X. (2020). Optimization of Enzyme-Assisted Extraction of Bioactive-Rich Juice from Chaenomeles Sinensis (Thouin) Koehne by Response Surface Methodology. J. Food Process. Preserv..

[7] Grassin C., Coutel Y. (2009). Enzymes in Fruit and Vegetable Processing and Juice Extraction. Enzymes in Food Technology.

[8] Wang W.-D., Xu S.-Y., Jin M.-K. (2009). Effects of Different Maceration Enzymes on Yield, Clarity and Anthocyanin and Other Polyphenol Contents in Blackberry Juice. Int. J. Food Sci. Technol..

[9] Ramadan M.F. (2018). Enzymes in Fruit Juice Processing.

[10] Sharma H.P., Patel H. (2017). Sugandha Enzymatic Added Extraction and Clarification of Fruit Juices—A Review. Crit. Rev. Food Sci. Nutr..

[11] Guo K. (2024). Changes in the Main Physicochemical Properties and Electrochemical Fingerprints in the Production of Sea Buckthorn Juice by Pectinase Treatment. Molecules.

[12] Figueiredo-González M., Cancho-Grande B., Simal-Gándara J. (2013). Effects on Colour and Phenolic Composition of Sugar Concentration Processes in Dried-on- or Dried-off-Vine Grapes and Their Aged or Not Natural Sweet Wines. Trends Food Sci. Technol..

[13] Zubaidi M.A., Czaplicka M., Kolniak-Ostek J., Nawirska-Olszańska A. (2023). Influence of Variety, Enzyme Addition and Destemming on Yield and Bioactive Compounds of Juices from Selected Hybrid Grape Varieties Cultivated in Poland. Foods.

[14] Sorrivas V., Genovese D.B., Lozano J.E. (2006). Effect of Pectinolytic and Amylolytic Enzymes on Apple Juice Turbidity. J. Food Process. Preserv..

[15] Bender A., de Souza A.L.K., Malgarim M.B., Caliari V., Kaltbach P., Costa V.B. (2021). Physicochemical and Sensory Properties of Grape Juices Produced from Different Cultivars and Extraction Systems. Semin. Agrar..

[16] Dutra M.d.C.P., Viana A.C., Pereira G.E., Nassur R.d.C.M.R., dos Santos Lima M. (2021). Whole, Concentrated and Reconstituted Grape Juice: Impact of Processes on Phenolic Composition, “Foxy” Aromas, Organic Acids, Sugars and Antioxidant Capacity. Food Chem..

[17] Puzovic A., Pelacci M., Simkova K., Hudina M., Rusjan D., Veberic R., Mikulic-Petkovsek M. (2024). Effect of Heat Pasteurization and Enzymatic Maceration on Yield, Color, Sugars, Organic Acids, and Phenolic Content in the ‘Merlot Kanthus’ Grape Juice. Beverages.

[18] Paixão N., Perestrelo R., Marques J.C., Câmara J.S. (2007). Relationship between Antioxidant Capacity and Total Phenolic Content of Red, Rosé and White Wines. Food Chem..

[19] Guler A. (2023). Effects of Different Maceration Techniques on the Colour, Polyphenols and Antioxidant Capacity of Grape Juice. Food Chem..

[20] Rinaldi M., Caligiani A., Borgese R., Palla G., Barbanti D., Massini R. (2013). The Effect of Fruit Processing and Enzymatic Treatments on Pomegranate Juice Composition, Antioxidant Activity and Polyphenols Content. LWT Food Sci. Technol..

[21] Gligor O., Mocan A., Moldovan C., Locatelli M., Crișan G., Ferreira I.C.F.R. (2019). Enzyme-Assisted Extractions of Polyphenols—A Comprehensive Review. Trends Food Sci. Technol..

[22] Dal Magro L., Goetze D., Ribeiro C.T., Paludo N., Rodrigues E., Hertz P.F., Klein M.P., Rodrigues R.C. (2016). Identification of Bioactive Compounds From Vitis Labrusca L. Variety Concord Grape Juice Treated With Commercial Enzymes: Improved Yield and Quality Parameters. Food Bioprocess Technol..

[23] (1999). Fruit and Vegetable Products. Preparation of Samples and Test Methods. Determination of Total Acidity.

[24] Oszmiański J., Kolniak-Ostek J., Lachowicz S., Gorzelany J., Matłok N. (2015). Effect of Dried Powder Preparation Process on Polyphenolic Content and Antioxidant Capacity of Cranberry (*Vaccinium macrocarpon* L.). Ind. Crops Prod..

[25] Kolniak-Ostek J. (2016). Chemical Composition and Antioxidant Capacity of Different Anatomical Parts of Pear (*Pyrus communis* L.). Food Chem..

[26] Re R., Pellegrini N., Proteggente A., Pannala A., Yang M., Rice-Evans C. (1999). Antioxidant Activity Applying an Improved ABTS Radical Cation Decolorization Assay. Free Radic. Biol. Med..

[27] Benzie I.F.F., Strain J.J. (1996). The Ferric Reducing Ability of Plasma (FRAP) as a Measure of “Antioxidant Power”: The FRAP Assay. Anal. Biochem..

[28] Yen G.-C., Chen H.-Y. (1995). Antioxidant Activity of Various Tea Extracts in Relation to Their Antimutagenicity. J. Agric. Food Chem..

[29] Nawirska-Olszańska A., Pasławska M., Stępień B., Oziembłowski M., Sala K., Smorowska A. (2020). Effect of Vacuum Impregnation with Apple-Pear Juice on Content of Bioactive Compounds and Antioxidant Activity of Dried Chokeberry Fruit. Foods.

[30] Kolniak-Ostek J., Oszmiański J. (2015). Characterization of Phenolic Compounds in Different Anatomical Pear (*Pyrus communis* L.) Parts by Ultra-Performance Liquid Chromatography Photodiode Detector-Quadrupole/Time of Flight-Mass Spectrometry (UPLC-PDA-Q/TOF-MS). Int. J. Mass Spectrom..

